# Confocal laser scanning and microCT evaluation of the intratubular penetration and sealing ability of calcium silicate and epoxy-resin endodontic sealers

**DOI:** 10.1007/s10266-026-01331-5

**Published:** 2026-02-06

**Authors:** Mario Alovisi, Vittorio Fenoglio, Elio Berutti, Francesca Fanelli, Allegra Comba, Andrea Baldi, Nicola Scotti, Damiano Pasqualini

**Affiliations:** Department of Surgical Sciences, Dental School, University of Turin, Via Nizza, 230, 10126 Turin, Italy

**Keywords:** Calcium–silicate sealer, MicroCT, Confocal laser scanning microscope, Sealing ability, Epoxy-resin sealer

## Abstract

This study aimed to compare the sealing ability of different calcium silicate-based and epoxy-resin endodontic sealers with single-cone cold technique through confocal laser scanning microscope (CLSM) and micro computed tomography (MicroCT) analysis. An in-vitro evaluation was conducted of voids and gaps occurring within the sealer and at its interfaces with the cone and canal walls, as well as of the penetration depth of the materials examined. Sixty-five single-rooted teeth with fully formed apex were collected. After root canal shaping the specimens were distributed in six different groups (*n* = 10) and a control group (*n* = 5): Group AH Plus Epoxy Resin Sealer (Dentsply Sirona), Group AH Plus Bioceramic Sealer (Dentsply Sirona), Group BC Sealer (Brasseler), Group BC Sealer HiFlow (Brasseler), Group BioRoot Flow Sealer (Septodont), group experimental sealer (Dentsply Sirona). All canals were sealed with single cone technique with the addition of a fluorescent dye. The specimens were MicroCT scanned and sectioned at four different levels of analysis for the CLSM evaluation. The differences between groups were analyzed with one-way ANOVA and post-hoc Bonferroni test (*P* < 0.05). Kruskall–Wallis and Mann–Whitney *U* tests were used for the non-parametric statistical analysis (*P* < 0.05). The microCT analysis reported an higher presence of voids and empty spaces for BioRoot Flow Sealer (Septodont) (*P* < 0.001). The CLMS imaging showed no statistical difference between the groups at the different points of analysis except for AH Plus Epoxy Resin Sealer (Dentsply Sirona) that showed a higher rate of empty spaces at the apical level (*P* = 0.044). No statistical differences were recorded for the total percentage of CLSM gap areas between groups. Interestingly, voids were most prevalent in the coronal and middle sections of all samples, corresponding to regions, where the canal is typically more oval (*P* = 0.03). Among the materials tested, the Experimental Sealer (Dentsply Sirona) achieved the deepest penetration into dentinal tubules (*P* = 0.038). The sealing performance of endodontic sealers used with the single-cone cold technique appears adequate and depends on both the properties of the sealer and the anatomy of the root canal.

## Introduction

The primary purpose of the endodontic treatment is the decontamination of the root canal system from bacteria, pulp residues, organic substrates and shaping debris while maintaining the root canal anatomy [1, 2]. Furthermore, the establishment of a hermetic seal through three-dimensional (3D) filling is mandatory to complete the endodontic disinfection [3, 4]. Therefore, the success of the endodontic treatment depends on an appropriate shaping, disinfection and filling of the root canal system [1–4].

Root canal filling plays a critical role in ensuring the long-term treatment success, as it prevents the bacterial leakage into the periapical tissue to cause disease [5]. Thus, emphasis is attributable to the endodontic sealer, that should be able to fill any gaps between the canal walls and the filling material [5]. Because gutta-percha does not adhere to root canal walls, endodontic sealer is mandatory to optimize the apical seal [6]. Biocompatibility, dimensional stability, radiopacity, flowability and low solubility are the main characteristics of an endodontic sealer, especially when it is used with a single cone cold technique [6, 7]. The unique properties of epoxy-resin and calcium silicate-based (CS) sealers allowed the clinicians to change the traditional lateral compaction and warm gutta-percha-based philosophies reinventing the “cold single-cone” technique [8, 9]. However, this technique may present drawbacks, including the use of a greater volume of sealer relative to gutta-percha and limited compaction of the sealer against the canal walls, which can lead to the formation of voids and gaps [8–10]. Thus, assessing sealers under single-cone cold obturation conditions is essential, as the sealer’s performance plays a crucial role in achieving an effective seal, regardless the obturation technique.

The CS sealers share similar compositions and properties with mineral trioxide aggregate (MTA), and offer additional benefits of being non-staining, having less impurities and smaller particle sizes. They are composed of alumina, zirconia, bioactive glasses, hydroxyapatites, calcium silicate and calcium phosphate [10]. In vitro studies have shown that CS sealers have excellent flowability, biocompatibility, adequate radiopacity, high pH, good dimensional stability and sealing ability [10]. They are also antibacterial and highly biocompatible with the ability to induce mineralization [11, 12]. Thus, CS sealers are bioactive cements containing and releasing calcium, which implies that the residual bacteria could be effectively walled inside dentinal tubules, undergoing a bactericidal action mediated by the high pH [12]. Moreover, the sealing performance of these materials may be enhanced by their capacity to expand during setting in the presence of water and moisture [13].

A recent survey showed that 60% of practitioners worldwide are currently using CS sealer-based obturation technique [14]. This technique is claimed to allow clinicians to obturate root canal systems using a simplified technique while maintaining high sealing ability and successful treatment outcome [14]. However, the sealing ability with this technique in different root canal anatomies is still debated. In addition, previous studies have noted that these materials are difficult to remove from the canals once set, and that they may fail to fully set in the absence of moisture within the root canal lumen [15, 16].

Resin-based sealers are widely used in endodontics due to their favorable physical–chemical properties: good flow and long setting time provide a proper sealer penetration inside dentinal tubules [17]. Currently, AH Plus epoxy resin sealer (Dentsply Sirona) is considered the gold standard for the comparisons in terms of sealing ability using a single cone cold technique [18] and it has been previously compared with CS sealers [19, 20]. However, new resin-based materials are being evaluated to enhance performance and address the limitations of CS sealers. A new experimental resin-based sealer has been introduced with optimized properties, such as flowability, setting time, and viscosity.

The quality of obturation achieved with epoxy-resin and CS sealers with cold single-cone technique may be similar compared with warm vertical compaction [21, 22]. However, no data are currently available on the filling quality of the new experimental resin-based sealer in comparison with other materials used for the single-cone cold technique. Moreover, no studies performed confocal laser scanning microscope (CLSM) and micro-computed tomography (MicroCT) analysis together to demonstrate the presence of voids and gaps after root canal filling with those sealers. Combining these methods provides complementary advantages: microCT allows three-dimensional visualization of empty spaces after filling, while CLSM offers highly detailed interfacial analysis of void location, area, and sealer penetration depth.

This in-vitro study aims to evaluate the sealing ability of different epoxy-resin and CS sealers through CLSM and microCT analysis. The null hypothesis was that no significant differences would be found among the tested sealers in terms of void formation and dentinal tubule penetration.

## Materials and methods

This manuscript has been written according to the Preferred Reporting Items for Laboratory studies in Endodontology (PRILE) 2021 [23]. The study was approved by the local Research Ethics Committee (Approval Code CS2_1127_2024).

### Samples selection

Sixty-five mandibular premolars with fully formed apex, similar root length and canal anatomy, without carious lesions and extracted for periodontal reasons were collected. The extracted teeth were donated for research purposes following written informed consent. The elements with previous endodontic treatment, significant calcifications, accentuated isthmuses, open wide apex or root resorptions were excluded, as well as elements presenting root fracture. A sample size of 10 per group was calculated with G*Power 3.1.4 (Kiel University, Kiel, Germany) to set the study power at 80% with ANOVA considering alpha level 0.05 (a large effect size of 1 was considered for the sample size computation). After the root debridement with Gracey curettes, the specimens were immersed in a 0.01% sodium hypochlorite (NaOCl) solution at 4 °C for 24 h (Niclor 5, OGNA, Muggiò, Italy) and then stored in saline solution until experimental use.

### Inclusion criteria

Specimens were placed on a custom holder and microCT scanned (SkyScan 1172 micro-CT, Bruker, Kontich, Belgium) to obtain overall information regarding the root canal anatomy to ensure the respect of the inclusion criteria. This first microCT scan was performed to outline the morphological parameters of the canals, and the following inclusion criteria were applied. Monoradicular fully formed root apices and canals measured 14 ± 2 mm from canal orifices to the apical foramen and showed < 20° primary root canal curvature in bucco-lingual projection according to the Schneider method [24]. The main curvature radius was selected ≤ 4 mm, with a point of maximum curvature located at the middle third. The proximal curvature was investigated from a mesio-distal projection and only teeth with a 10°–20° primary curvature were considered [25]. Teeth with significant root canal calcifications were excluded. Teeth not concurring with the inclusion criteria regarding canal curvature and patency were excluded.

### Samples preparation

After access cavity preparation, the manual canal scouting with a K-File #10 (Dentsply Maillefer) was performed in all the specimens up to working length (WL). The WL was determined under 10X magnification (OPMI Pro Ergo, Carl Zeiss, Oberkochen, Germany) when the tip of the instrument was visible at the apical foramen. For all specimens, the mechanical glide path was completed with nickel–titanium (NiTi) rotary ProTaper Ultimate Slider (Dentsply, Maillefer) using an endodontic motor (X-Smart Plus, Dentsply Maillefer) with 16:1 contra angle at 300 rpm, 4 Ncm at full WL. Then, the shaping was performed with ProTaper Ultimate (PTU) (Dentsply, Maillefer) up to F2 (25/0.08). The instruments were removed and cleaned each time after three pecking motions until WL was reached. The irrigation was ensured alternating 5% NaOCl (Niclor 5, OGNA, Muggiò, Italy) and 10% EDTA (Tubuliclean, OGNA, Italy) for a total of 5 ml for each solution using a 30G needle up to the apical third [26]. The first and the last irrigation was performed with NaOCl and the recapitulation was ensured with a size 10 K-File between each instrument [27]. The procedures were led by single expert operator with more than 10 years of practice in endodontics who avoided intentional brushing movements. Afterward, the root canals were dried with absorbent sterile paper points and a second microCT scan was made at high resolution for post-shaping analyses.

Then the canals were randomly assigned to six different groups (*n* = 10) and a control group (*n* = 5) according to the filling technique using a computer-generated randomization system. The samples were filled with cold single cone technique with different sealers for each group and each sealer was labeled with a fluorescent dye (Fluo 3 Invitrogen—https://www.thermofisher.com/order/catalog/product/F1241) at an approximate concentration of 0.1% to allow CLSM visualization [26–29]. The groups were divided as follows:

Group AH Plus Epoxy Resin Sealer (Dentsply Sirona), Group AH Plus Bioceramic Sealer (Dentsply Sirona), Group BC Sealer (Brasseler), Group BC Sealer HiFlow (Brasseler), Group BioRoot Flow Sealer (Septodont), group experimental sealer (Dentsply Sirona) and single cone technique plus negative control with the use of non-fluorescent-labeled AH Plus Bioceramic Sealer to set the fluorescent threshold for CLSM analysis. In all groups, a dedicated gutta-percha point (F2 conform fit—Dentsply Sirona) was selected and used with the sealer according to manufacturer’s instructions. All specimens were stored at 37 C° in physiological solution for 1 week to obtain a complete set of the sealer. Coronally, they were sealed with a provisional restoration (Cavit, 3 M ESPE) to simulate the clinical conditions. Afterward, the samples were microCT scanned at high resolution before sectioning and CLSM analysis. MicroCT analysis was used to assess the three-dimensional volume of gaps and voids after obturation, whereas CLSM analysis was employed to examine two-dimensional cross sections at different levels under high magnification and to measure sealer penetration depth.

### MicroCT analysis

MicroCT analysis was performed with SkyScan 1172 Bruker (Kontich, Belgium). The third microCT scan was performed after filling to compare the volume of the obturation material. Each sample was scanned at high resolution with the following microCT parameters: 100 kV and 100 μA, isotropic resolution equal to 20.86 μm/pixel, in a timeframe of about 2 h for each sample. Afterward, the scans are aligned through the software Data Viewer (Bruker, Kontich, Belgium) to compare the pre- and post-filling volumes. The volumetric analysis was performed with CTAn software (Bruker, Kontich, Belgium) and the percentage of voids inside the root canal space for each sample was evaluated. The analysis was made through the difference between the volume of the empty canal and the volume of the obturation material: the percentage score zero indicates a perfect 3D obturation.

### CLSM analysis

The root of each element was sectioned perpendicularly to the long axis of the tooth, at the level of the cementum–enamel junction (CEJ). The samples were horizontally sectioned perpendicular to the root canal axis creating disks with 1 mm thickness. The horizontal sections were distributed as follows: Apical 1 (A1) 2 mm from the apical foramen, Apical 2 (A2) 4 mm from the apical foramen, Middle (M) 7 mm from the apical foramen and Coronal (C) 11 mm from the apical foramen. This distribution was created to allow a direct comparison between 2D CLSM and MicroCT slices for each level of analysis.

The fluorescent staining was performed with Fluo-3 fluorescent dye (Invitrogen—https://www.thermofisher.com/order/catalog/product/F1241) due to its ability to bind bioceramic calcium ions and epoxy resin sealer’s calcium tungstate [28, 29]. Imaging was performed using a Leica SP8 confocal system (Leica Microsystems) equipped with an argon ion and a 561 nm DPSS lasers. Samples were imaged using a HCX PL APO 20x/0.75 NA objective. Images were obtained using a 10X objective and performing 20 μm Z-stacks with 1 μm step size and evaluated with Image J software [30]. Images were then analyzed with ImageJ software (NIH, Bethesda, MD, USA) [31]. It was fundamental to discriminate the root canal voids by the gutta-percha cone. Therefore, to enhance the visualization and the analysis, different look up tables (LUT) were applied using ImageJ software (NIH, Bethesda, MD, USA). The percentage of empty spaces and the mean depth of sealer penetration inside dentinal tubules was recorded for each point of analysis [27].

### Statistical analysis

The analyses were performed by the same blinded operator, and the differences were analyzed with one-way ANOVA and post-hoc Bonferroni test (*P* < 0.05). The one-way ANOVA with Bonferroni post-hoc test was selected for variables that met the assumptions for parametric analysis, as these tests allow comparison of mean differences among multiple groups. Normality was assessed using the Shapiro–Wilk test. For data sets that did not satisfy these assumptions, non-parametric Kruskall–Wallis and Mann–Whitney *U* tests were applied.

## Results

### MicroCT analysis

There was no incidence of instrument fracture during canal preparation. Canal volumes, surface areas and mean apical diameters at baseline are presented in Table 1. The values displayed preoperative homogeneity between groups (*P* > 0.05).

**Table 1:** Sample baseline characteristics in all groups (mean, STD). Statistical significance indicated by *P* < 0.05. *Apical diameters (mean ± SD) at 1 mm from apical foramen

	AH Plus Epoxy Resin Sealer (Dentsply Sirona)	AH Plus Bioceramic Sealer (Dentsply Sirona)	BC Sealer (Brasseler)	BC Sealer HiFlow (Brasseler)	BioRoot Flow Sealer (Septodont)	Experimental sealer (Dentsply Sirona)	*P*
Canal volumes (mm^3^)	3.09 ± 0.67	2.76 ± 0.87	2.39 ± 0.92	2.57 ± 1.09	2.66 ± 1.91	2.71 ± 1.12	0.56
Canal surface area (mm^2^)	19.32 ± 2.87	20.15 ± 3.05	18.09 ± 2.37	17.21 ± 3.17	19.91 ± 2.87	21.01 ± 4.07	0.19
Apical diameters* (mm)	0.21 ± 0.02	0.23 ± 0.4	0.19 ± 0.19	0.20 ± 0.08	0.22 ± 0.38	0.24 ± 1.78	0.13

No differences were described between the microCT pre-operative volumes indicating homogeneity between groups (*P* = 0.569). The difference between the microCT pre- and post-filling volumes showed no statistically significant differences between the groups, except for the Group BioRoot Flow Sealer (Septodont) that showed significantly different results regarding the following parameters (Table 2):

- MicroCT object volume DELTA (µm^3^) (*P* = 0.002): this indicates that the difference between pre- and post-filling root canal volumes is significantly higher for the group BioRoot Flow Sealer (Septodont) compared with the other groups. This suggests a higher presence of voids inside the root canal after filling.
- MicroCT object volume DELTA (%) (*P* = 0.001): this indicates that the difference expressed in rates between pre- and post-filling root canal volumes is significantly higher for the group BioRoot Flow Sealer (Septodont) compared with the other groups. This suggests a higher discrepancy between pre- and post-operative volumes and a higher presence of empty spaces.

**Table 2:** *P* values for each one-to-one comparison between tested sealers for different parameters are reported. The BioRoot Flow Sealer is compared with others

Groups	MicroCT object volume DELTA (μm^3^)	MicroCT object volume DELTA (%)
BioRoot Flow Sealer vs AH Plus Epoxy Resin Sealer	*P* = 0.008	*P* = 0.001
BioRoot Flow Sealer vs AH Plus Bioceramic Sealer	*P* = 0.007	*P* = 0.001
BioRoot Flow Sealer vs BC Sealer	*P* = 0.004	*P* = 0.001
BioRoot Flow Sealer vs BC Sealer HiFlow Sealer	*P* = 0.004	*P* = 0.001
BioRoot Flow Sealer vs Experimental sealer	*P* = 0.008	*P* = 0.001

Thus, the BioRoot Flow Sealer (Septodont) group exhibited a greater number of voids and empty spaces within the filled root canals, whereas the other tested materials showed comparable results.

### CLSM analysis

The CLSM evaluation of the rate of empty space for each group was divided in different points of analysis (Coronal, Middle, Apical 2, Apical 1).

- *Point of analysis Coronal (C)* There were no statistically significant differences between groups that showed a similar rate of empty spaces after filling (*P* = 0.221).
- *Point of analysis Middle (M)* There were no statistically significant differences between groups (*P* = 0.234). This implies that all sealers filled similarly the middle third of the evaluated samples, even if a higher variability of the results was reported for the Group AH Plus Epoxy Resin Sealer (Dentsply Sirona) compared with the others.
- *Point of analysis Apical 2 (A2)* There were no statistically significant differences between groups (*P* = 0.123). All sealers filled similarly the A2 point of analysis of the evaluated samples. A higher variability of the results was reported for the Group AH Plus Bioceramic Sealer (Dentsply Sirona) and the group experimental sealer (Dentsply Sirona) compared with the others.
- *Point of analysis Apical 1 (A1)* There were no statistically significant differences between groups, except for the group AH Plus Epoxy Resin Sealer (Dentsply Sirona) that showed a higher rate of empty spaces compared with the other groups (*P* = 0.019).

**Fig. 1:**
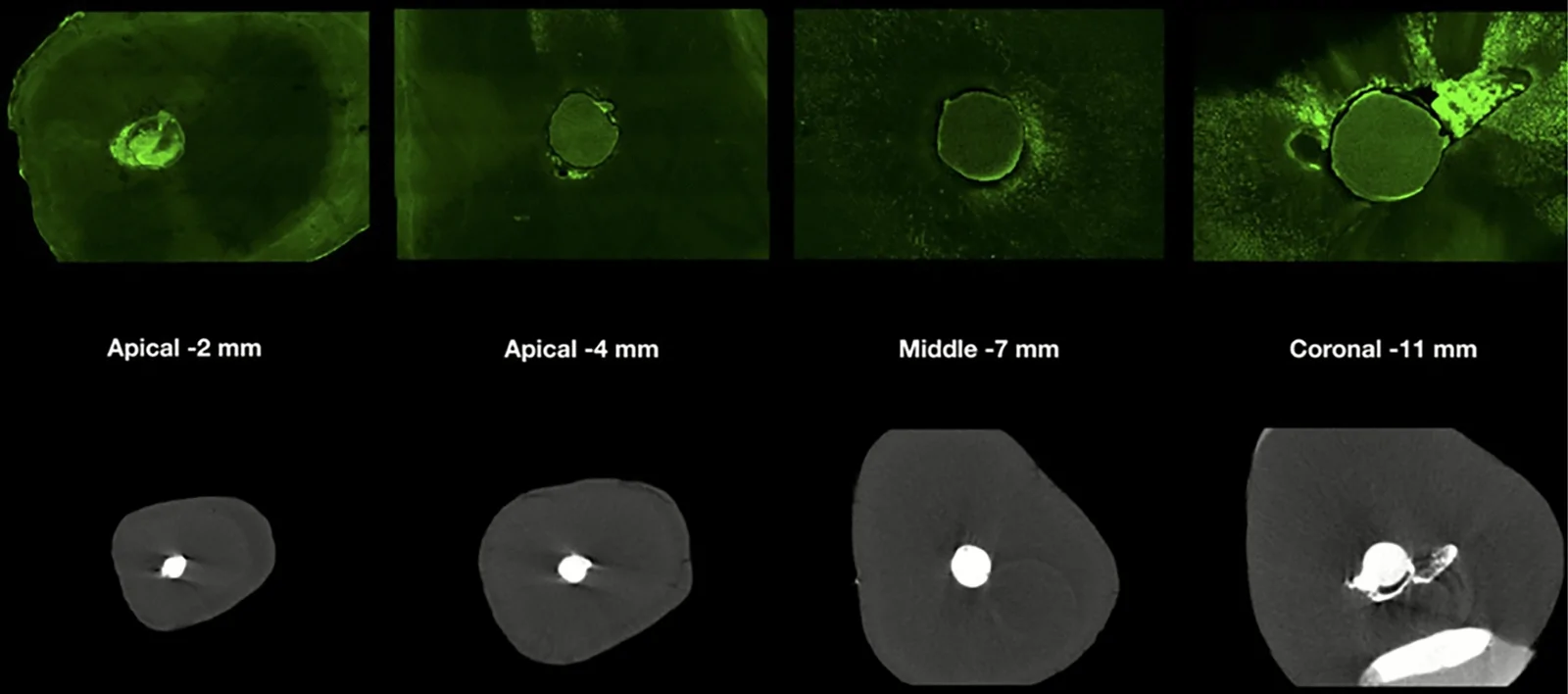
MicroCT and CLSM analysis showed that the higher percentage of voids was reported In the C and M points of analysis (*P* = 0.03)

Therefore, the CLSM analysis revealed statistically significant differences for the AH Plus Epoxy Resin Sealer (Dentsply Sirona), which exhibited a greater number of voids and empty spaces in the apical portion compared with the other materials, among which no differences were observed. The analysis of the total percentage of CLSM gap areas in all points of analysis together (C, M, A1 and A2) reported no statistically significant differences between groups (*P* = 0.238) (Table 3). However, among all samples the higher percentage of voids was reported in the C and M points of analysis (*P* = 0.031) (Fig. 1).

**Table 3:** Analysis of the total percentage (± SD) of CLSM gap areas in all points of analysis (C, M, A1 and A2) reported no statistically significant differences between groups (*P* = 0.238)

Groups	Total CLSM gap area (%)
AH Plus Epoxy Resin Sealer (Dentsply Sirona)	15.46 ± 5.49
AH Plus Bioceramic Sealer (Dentsply Sirona)	16.31 ± 8.61
BC Sealer (Brasseler)	18.51 ± 3.35
BC Sealer HiFlow (Brasseler)	14.32 ± 5.07
BioRoot Flow Sealer (Septodont)	19.41 ± 3.29
Experimental Sealer (Dentsply Sirona)	15.87 ± 3.86

**Fig. 2:**
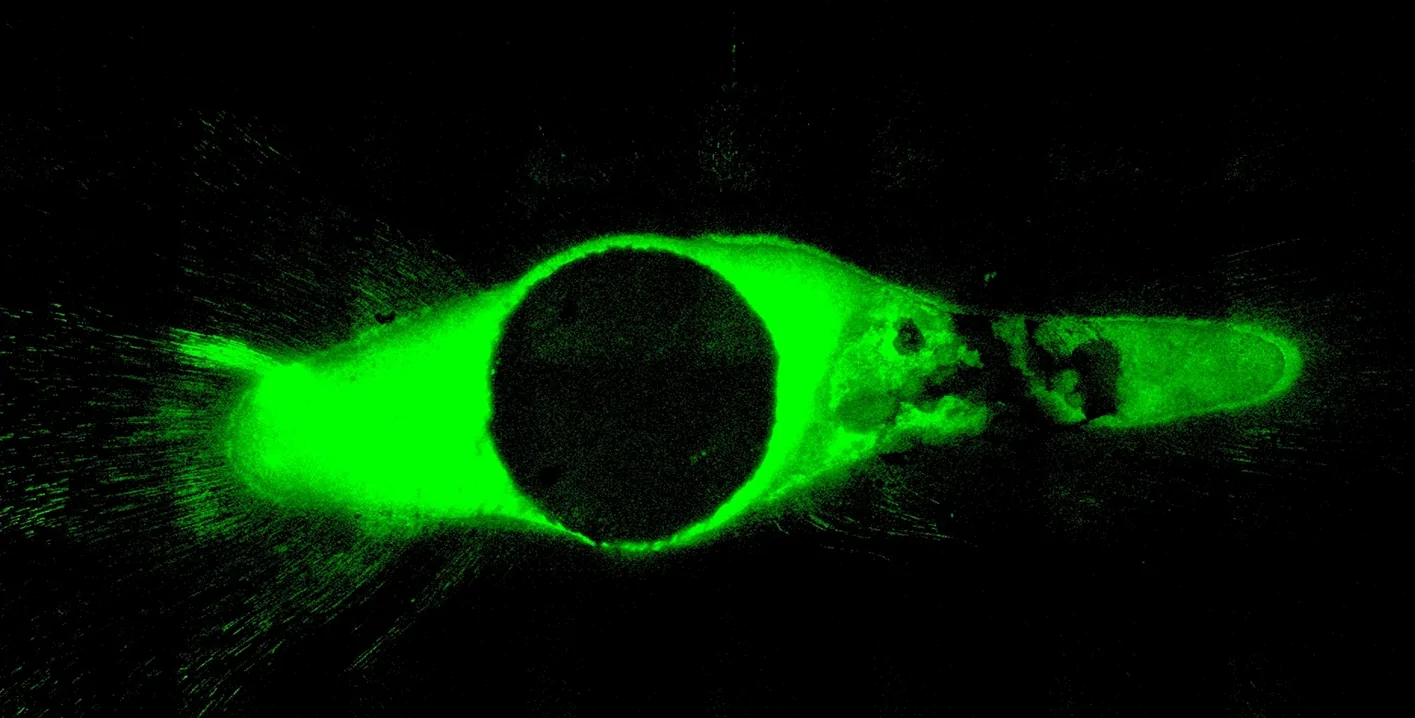
Confocal laser scanning microscope image displaying the penetration depth of a tested sealer in the dentine tubules of the coronal third of a root canal. The deepest penetration value and the sealing ability is highly influenced by the canal geometry

Using CLSM, the mean depth of penetration of the sealers into the dentin tubules was evaluated. The deepest penetration value was not reported because highly influenced by the canal geometry (Fig. 2). The mean penetration depth, calculated in pixels, for each slice of each tested sealer is represented in Table 4. There were no statistical differences for each group and for any point of analysis (*P* > 0.05), except for the Group AH Plus Bioceramic Sealer (Dentsply Sirona) that showed the lower penetration depth in the coronal third (*P* = 0.042) and the group experimental sealer (Dentsply Sirona) that presented the deepest intratubular penetration compared to other groups, especially in the coronal portion (*P* = 0.038).

**Table 4:** Mean sealer penetration depth (pixels) for each slide of the tested sealers (mean, ± STD) analyzed through CLSM images. There were no statistical differences for each group and for any point of analysis (*P* > 0.05), except for the Group AH Plus Bioceramic Sealer (Dentsply Sirona) that showed the worst penetration depth in the coronal third (*P* = 0.042) and the group experimental sealer (Dentsply Sirona) that presented the deepest intratubular penetration compared to other groups, especially in the coronal part (*P* = 0.038). * Statistically significant values (*P* < 0.05)

Groups	Coronal part (11 mm from apex)	Middle part (7 mm from apex)	Apical (4 mm from apex)	Apical (2 mm from apex)
AH Plus Epoxy Resin Sealer (Dentsply Sirona)	87.45 ± 28.86	83.89 ± 40.09	55.87 ± 37.98	52.05 ± 29.45
AH Plus Bioceramic Sealer (Dentsply Sirona)	46.30 ± 30.10*	40.09 ± 23.46	32.97 ± 24.83	32.48 ± 21.25
BC Sealer (Brasseler)	71.86 ± 33.09	55.23 ± 23.11	51.24 ± 19.20	46.65 ± 25.62
BC Sealer HiFlow (Brasseler)	77.98 ± 40.76	66.49 ± 41.24	36.04 ± 12.51	54.58 ± 37.82
BioRoot Flow Sealer (Septodont)	83.27 ± 42.12	64.80 ± 42.86	49.38 ± 30.25	21.73 ± 12.90
Experimental Sealer (Dentsply Sirona)	103.22 ± 32.86*	81.92 ± 31.24	59.55 ± 28.73	49.40 ± 19.04

## Discussion

The results reported that the tested sealers provided an adequate 3D filling of the endodontic space, even if this outcome seemed influenced by the root canal anatomical characteristics. However, the results are consistent with the current scientific literature [32].

Compared to traditional warm gutta-percha, the epoxy-resin and CS sealer-based single-cone cold technique is deemed to be easier, faster and cost-effective [32]. In the single-cone cold technique, a pre-fitted gutta-percha point is inserted into the canal space filled of sealer to create hydraulic pressure, driving a three-dimensionally obturation without compaction [8, 9]. However, it is critical to have sufficient sealer in the canal with an adequate distribution to prevent voids formation [8].

Previous studies reported that the sealing ability of the CS and epoxy resin sealers with single cone cold technique showed similar results [33]. Otherwise, other reports claimed differences among these two materials regarding the sealing ability [34]. In addition, no studies are available on the new resin-based experimental sealer with new physical properties. In this study, the microCT scans and volume reconstruction appeared mandatory to adequately evaluate the 3D root canal anatomy and to ensure a pre-operative homogeneity between the samples. Moreover, they allowed a standardization of the points of analysis for the CLSM imaging. The combination of microCT and CLSM data was considered an extremely accurate imaging method for the endodontic filling materials [35, 36].

Some studies investigated sealing ability and dentinal tubule penetration of calcium silicate and epoxy resin-based sealers through CLSM using Rhodamine B as a fluorophore [35, 36]. However, because of the hydrophilic nature of calcium silicate-based sealers, the results of Rhodamine B dentin penetration could be overestimated due to false-positive findings [37]. Therefore, the Fluo-3 (Invitrogen, Carlsbad) staining was proposed to trace CS sealers penetration inside dentinal tubules, due to its ability to bind bioceramic calcium ions and epoxy resin sealer’s calcium tungstate [29, 37, 38]. Furthermore, although previous studies reported adequate sealing ability of sealers used with the single-cone cold technique, they did not include both microCT and CLSM analyses nor did they consider the oval shape of root canals [32–37].

In this study, the trend obtained from the microCT volumes have been confirmed by the CLSM examination, even if statistically significant results were not duplicated. This may be due to the difference between 3D (MicroCT) and 2D (CLSM) imaging. However, the CLSM is considered extremely more accurate than microCT for the 2D analysis of root canal sections and it is mandatory for the accurate analysis of the presence of voids and gaps in the axial sections [39].

The combination of 2D and 3D imaging techniques showed that the higher percentage of voids was in the coronal and middle root canal portions. The root canals are anatomically ovoidal especially in the coronal and middle third. This may be detrimental for the single cone cold technique regardless the sealer used, and this method could not ensure a proper sealer compaction due to a reduction of the forces directed against canal walls. Therefore, clinically it may be important to discriminate the use of the single cone cold technique basing on the canal anatomy, where large and oval canals could be less indicated for this methodology. Moreover, the use of highly flowable sealers may trap air bubbles and voids both between the cone, the sealer and the canal walls. In addition, the single cone cold technique, regardless the sealer used, may be operator dependent [39]. The operator skills may be crucial for the quality of the root canal filling, more than with other techniques.

In this study, CLSM analysis reported that AH Plus Epoxy Resin Sealer created more empty spaces in the apical root canal third compared with the CS sealers. Otherwise, the experimental sealer (Dentsply Sirona), that has a resin composition and an increased viscosity and ability to adhere to the gutta-percha cone, displayed similar characteristics compared with the CS sealers. It seemed able to ensure a better sealing in the apical portion compared to AH Plus Epoxy Resin Sealer, reaching the quality standards of the CS sealers. For instance, the AH Plus Bioceramic Sealer (Dentsply Sirona) and the experimental sealer (Dentsply Sirona) showed similar behaviour both in the CLSM and microCT analysis, while the experimental sealer showed less variability for all points of analysis. In addition, it was able to penetrate deeper inside dentinal tubules compared to the other tested materials. Nevertheless, the overall comparison between CS sealers showed that the BioRoot Flow Sealer (Septodont) seemed to create more empty spaces in the microCT volumes. This outcome may be influenced by the physical and structural characteristics of the sealer itself that appear fundamental, more than usually happens with warm vertical gutta-percha compaction [40].

The ultimate criteria used to determine whether a new material or technique is appropriate for clinical application is the clinical success [41]. Clinical studies evaluating the outcomes of obturation are typically challenging to do due to the inherent difficulties in controlling other confounding variables, such as the patient’s systemic conditions, pre-op diagnosis, instrumentation and irrigation protocols, and the quality of coronal restoration [41]. As a result, few randomized controlled trials are available [42–45]. However, the analysis regarding clinical success demonstrated comparable outcomes comparing single cone cold techniques to warm vertical gutta-percha obturation [46, 47].

The limits of this study include the number of the samples and the anatomical variability of the root canal anatomies. Moreover, it is important to remember that the sealer penetration depth inside the dentinal tubules does not directly influence the clinical endodontic success. Therefore, most of the analysis should be directed through the evaluation of the gaps inside the canal: voids and empty spaces may accommodate residual bacteria that could be able to replicate compromising the outcomes. Therefore, more in-vitro and in-vivo studies are needed to confirm the sealing ability and the long-term success of these materials.

## Conclusion

The combined microCT and CLSM analysis allowed a detailed description of the sealing ability of the endodontic sealers used with single cone cold technique. All tested sealers showed acceptable sealing ability although with some variations resulting from their composition and physical properties. Specifically, the BioRoot Flow Sealer (Septodont) group exhibited significantly more empty spaces in the microCT analysis, while the CLSM evaluation revealed a higher number of apical voids for the AH Plus Epoxy Resin Sealer (Dentsply Sirona). The Experimental Sealer (Dentsply) demonstrated greater penetration depth than the other materials tested. Regardless of the sealer’s properties, the root canal anatomy—often ovoid, particularly in the coronal and middle thirds—may negatively affect sealing ability when the single-cone cold technique is used.

## Data Availability

The data that support the findings of this study are available from the corresponding author upon reasonable request
